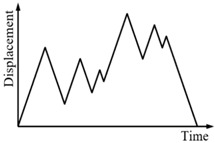# Numerical Simulation and Experimental Study of a Simplified Force-Displacement Relationship in Superelastic SMA Helical Springs

**DOI:** 10.3390/s19010050

**Published:** 2018-12-23

**Authors:** Bin Huang, Hongwang Lv, Yang Song

**Affiliations:** School of Civil Engineering and Architecture, Wuhan University of Technology, Wuhan 430070, China; lv_hongwang@whut.edu.cn (H.L.); song_yang@whut.edu.cn (Y.S.)

**Keywords:** SMA helical spring, superelasticity, force-displacement relationship, multilinear constitutive model, tension test, hysteresis, self-sensing

## Abstract

This paper proposes a new force-displacement model for superelastic shape memory alloy (SMA) springs under complex loading and unloading. For the SMA wires used to make superelastic springs, a new multilinear constitutive model based on a modification of the 1D Motahari model is developed. In the modified model, the stress-strain relation curves are changed to fit the experimental results. Furthermore, the established force-displacement relationship of the springs considers the impact of not only the torque but also the moment on the cross sections of the SMA wires. Afterwards, a series of tension tests are performed on four NiTi helical spring specimens under various loading conditions. From the numerical simulations and experimental results, it is shown that, compared with the force-displacement curves for the SMA springs simulated by the Motahari model, those simulated by the proposed model can better approximate the experimental results. The new model inherits the advantage of simple computation of the multilinear constitutive model and can predict the force-displacement relation for superelastic SMA springs very well. Furthermore, due to the self-sensing properties of the SMA springs, the new model is very significant for establishing a new strategy for measuring the displacements or forces of SMA springs under complex loading and unloading.

## 1. Introduction

A shape memory alloy (SMA) is a smart material that has not only unique shape memory and superelastic effects but also good damping characteristics, fatigue resistance and corrosion resistance. SMAs are widely used in the aerospace industry, robotics, and biomedical and other fields [1,2,3,4,5]. Superelastic SMAs possess a hysteretic stress-strain relationship [6,7,8], which results in their inherent energy dissipation capacity. With their energy dissipation capacity and self-centering property, superelastic SMAs have been actively researched in recent years in civil engineering for structural vibration control [9,10,11,12,13,14,15,16]. On the other hand, martensite SMAs with shape memory effects have been researched for repairing concrete structures [17,18]. The helical spring is an important form of SMAs, and research on SMA helical springs and their application to seismic resistance in civil engineering structures has attracted the attention of researchers. For example, the SMA helical spring is combined with a conventional damper or an isolation bearing to form a passive damping system, which uses superelasticity to provide a restoring force and dissipate seismic input energy, thereby achieving control of the structural vibration responses [19,20,21,22,23]. To further understand the superelastic effect in an SMA helical spring, it is very important to study the mechanical properties of an SMA helical spring under complex loading and unloading conditions. In addition, motivated by the fact that an SMA device can also be used as a sensor by monitoring its electric resistance or inductance change [24,25,26,27,28,29,30], one can develop the self-sensing functions of SMA springs to measure the displacements or forces of springs under a seismic load. These self-sensing functions are also very significant for understanding the mechanical properties of SMA springs subjected to complex loading.

At present, researchers mainly study the mechanical properties of SMA springs based on the complex thermodynamic stress-strain constitutive model of SMA materials in a superelastic state. For example, Tobushi and Tanaka [31] proposed the relationship between the load and deformation of an SMA helical spring based on the stress-strain-temperature model of SMA materials proposed by Tanaka [32]. Liang and Rogers [33] established the force-displacement relationship for an SMA helical spring based on the thermodynamic constitutive model proposed by Liang and Rogers [34]. Toi et al. [35] used the one-dimensional constitutive model [36] to perform finite element analysis on the superelastic large deformation behavior of an SMA helical spring using linear Timoshenko beam elements. Aguiar et al. [37] used the constitutive model of SMA materials proposed by Paiva et al. [38] to study the superelasticity and shape memory effect of an SMA helical spring. Mirzaeifar et al. [39] performed a numerical analysis of the mechanical response of a superelastic SMA helical spring under an axial force based on their previous works [40,41]. Based on the model proposed by Chemisky et al. [42], Thiebaud and Zineb [43] explored the damping effect of a superelastic SMA helical spring by the equivalent complex stiffness. Enemark et al. [44] used the modified Brinson’s model to theoretically analyze and experimentally verify the mechanical properties of an SMA helical spring. Savi et al. [45] considered the influence of geometric nonlinear size on the mechanical behavior of an SMA helical spring based on the one-dimensional constitutive model proposed by Auricchio et al. [46]. Mehrabi and Ravari [47] studied the force-displacement relationship for a superelastic SMA helical spring at room temperature using the microplane model proposed by Mehrabi and Kadkhodaei [48]. However, only single-cycle loading is considered in these papers, and complex loading cases are not studied. In general, to establish the force-displacement relationship for an SMA helical spring, it is required to solve a large number of nonlinear thermodynamic equations in the stress-strain-temperature model. This process complicates the simulation of the force-displacement relationship.

To improve the simulation efficiency of the force-displacement relationship, a multilinear constitutive model of an SMA material was proposed by Motahari and Ghassemieh [49] to replace the nonlinear function models driven by solving the nonlinear thermodynamic equations. Inspired by this idea, this paper proposes a new mechanical model of SMA springs based on a modification to the 1D model proposed by Motahari and Ghassemieh. In the modified model, the stress-strain relationships, e.g., the subloops, of the SMA material are changed to fit the experimental results. Furthermore, the established force-displacement relationship of the springs considers the impact of not only the torque but also the moment on the cross sections of the SMA wires. Afterwards, a series of tension tests are conducted on four NiTi helical spring specimens under various loading cases. The numerical results are compared with the experimental results to verify the validity and correctness of the proposed model. Meanwhile, it is found that compared with the force-displacement curves simulated for the SMA springs by the Motahari model, those simulated by the proposed model are closer to the experimental results. Furthermore, using self-sensing characterizations, such as the displacement-resistance relationship and the displacement-coil inductance relationship for the SMA springs, this new model will be very useful for providing a new strategy to measure the displacements or forces of SMA springs.

## 2. Mechanical Model of a Superelastic SMA Helical Spring

### 2.1. Multilinear Constitutive Model of SMA Material

From previous experiments, it was found that under complex loads, the force-displacement relationship curves of SMA springs basically consist of major loops and subloops. This characteristic is closely related to the stress-strain-temperature relationship of an SMA wire material, which exhibits major loops and subloops that also exist under complex loading and unloading processes. Therefore, it is necessary for us to first develop a mechanical model for SMA wire material to include the major loops and subloops.

#### 2.1.1. Major Loop

**Figure 1:**
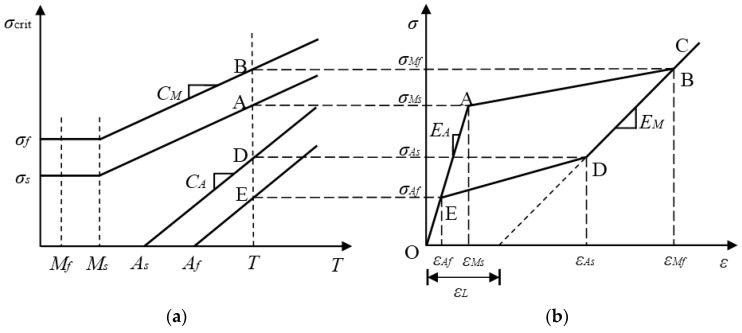
Schematic of the major loop in the multilinear constitutive model: (**a**) critical stress versus temperature; (**b**) superelastic stress-strain behavior.

Using the normal stress-strain multilinear constitutive model of SMA material proposed by Motahari and Ghassemieh [49] and considering the reorientation of different martensite variants [36], the major loop in the multilinear constitutive model can be obtained, as shown in Figure 1. In Figure 1a, the *σ*_crit_ axis is the critical stress axis, and the *T* axis is the temperature axis.

Before describing the stress-strain path of the major loop, four linear relationships between the four critical stresses *σ_Ms_*, *σ_Mf_*, *σ_As_* and *σ_Af_* and the ambient temperature *T* should be established. Note that this paper only considers the superelastic properties of an SMA material whose temperature is not less than *A_f_*. According to Figure 1a, these linear relationships can be represented by the following formulas

(1) $$\sigma_{M_{s}}=\sigma_{s}+C_{M}(T-M_{s})$$

(2) $$\sigma_{M_{f}}=\sigma_{f}+C_{M}(T-M_{s})$$

(3) $$\sigma_{A_{s}}=C_{A}(T-A_{s})$$

(4) $$\sigma_{A_{f}}=C_{A}(T-A_{f})$$

where *M_s_* and *M_f_* are the temperatures at the beginning and end of the martensitic transformation, respectively; *A_s_* and *A_f_* are the temperatures at the beginning and end of the austenitic transformation, respectively; *T* is the ambient temperature; *C_M_* and *C_A_* are the material parameters related to the martensite and austenite, respectively, or the slopes of the lines in Figure 1a; *σ_s_* and *σ_f_* are the starting and final critical stresses, respectively, while the SMA material transforms from twinned martensite to detwinned martensite; *σ_Ms_* and *σ_Mf_* are the critical stresses at the beginning and end of the martensitic transformation, respectively; and *σ_As_* and *σ_Af_* are the critical stresses at the beginning and end of the austenitic transformation, respectively.

In Figure 1b, the normal stress-strain curve O-A-B-C-D-E-O is a major loop, and from the austenitic phase to the martensitic phase, a complete phase transformation of the SMA material occurs. The entire path of the major loop contains four linear segments, which are described as follows:

Paths O-A and E-O (elastic-fully austenite):

(5) $$\sigma =E_{A}\varepsilon$$

Path A-B (forward transformation):

(6) $$\sigma =\sigma_{M_{s}}+\frac{\sigma_{M_{f}}-\sigma_{M_{s}}}{\varepsilon_{M_{f}}-\varepsilon_{M_{s}}}(\varepsilon -\varepsilon_{M_{s}})$$

Paths B-C and C-D (elastic-fully martensite):

(7) $$\sigma =\sigma_{M_{f}}+E_{M}(\varepsilon -\varepsilon_{M_{f}})$$

Path D-E (reverse transformation):

(8) $$\sigma =\sigma_{A_{s}}+\frac{\sigma_{A_{f}}-\sigma_{A_{s}}}{\varepsilon_{A_{f}}-\varepsilon_{A_{s}}}(\varepsilon -\varepsilon_{A_{s}})$$

where *E_A_* and *E_M_* are the SMA’s Young’s moduli of austenite and martensite, respectively; *ε_L_* is the maximum residual strain; *ε_Ms_* and *ε_Mf_* denote the starting and final critical strains of the martensitic transformation, respectively; and *ε_As_* and *ε_Af_* are the starting and final critical stresses of the austenitic transformation. Furthermore, the relationships between the critical stresses and strains can be expressed as:

(9) $$\varepsilon_{M_{s}}=\frac{\sigma_{M_{s}}}{E_{A}}$$

(10) $$\varepsilon_{M_{f}}=\varepsilon_{L}+\frac{\sigma_{M_{f}}}{E_{M}}$$

(11) $$\varepsilon_{A_{s}}=\varepsilon_{L}+\frac{\sigma_{A_{s}}}{E_{M}}$$

(12) $$\varepsilon_{A_{f}}=\frac{\sigma_{A_{f}}}{E_{A}}$$

It should be noted that since the multilinear constitutive model is taken in this paper, the nonlinearities observed at the beginning and end of the forward and reverse martensitic transformations are not completely considered, which means that the stress-strain curves are not smooth in these places. However, this characteristic makes the calculation of the model easier than that of the models used in the literature, such as Lagoudas et al. [50]. 

#### 2.1.2. Subloops

**Figure 2:**
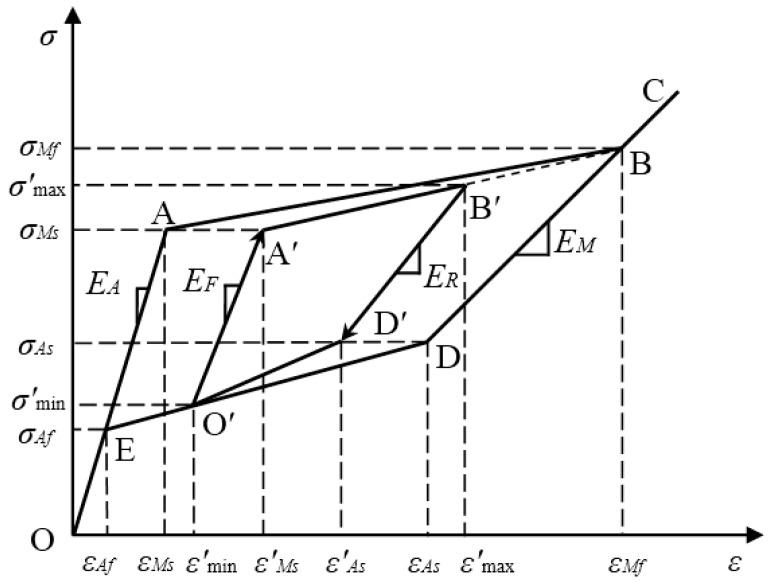
Schematic of the hysteresis curve model of the subloop.

When the incomplete phase transformation of the SMA material occurs, the constitutive model of the subloop hysteresis can be described by the multilinear curve O′-A′-B′-D′-O′, as shown in Figure 2. In Figure 2, the elastic modulus of the linear path O′-A′ or B′-D′ is different from those of the austenite and martensite phases, and is regarded as a function of martensite volume fraction as [51]

(13) $$\frac{1}{E_{i}(\xi )}=\frac{1-\xi }{E_{A}}+\frac{\xi }{E_{M}}\;(i=\;F,\;R)\;$$

where *ξ* is the martensite volume fraction, and the symbol *F* and *R* indicate the loading and unloading case, respectively.

For the path O′-A′ in the reloading case, considering the linear relationship between the transformation strain and the martensite volume fraction [49,52], there is

(14) $$\xi =\frac{\varepsilon_{\min}^{\prime }-\varepsilon_{A_{f}}}{\varepsilon_{A_{s}}-\varepsilon_{A_{f}}}$$

In the case of unloading, it is assumed that the martensite volume fraction can be calculated by

(15) $$\xi =\frac{\varepsilon_{M_{f}}-\varepsilon_{\max}^{\prime }}{\varepsilon_{M_{f}}-\varepsilon_{M_{s}}}$$

where $\varepsilon_{\min}^{\prime }$ and $\varepsilon_{\max}^{\prime }$ indicate the minimum strain and the maximum strain in the subloop, respectively.

According to Equations (13)–(15), the elastic moduli of the paths O-A′ and B′-D′ of the subloop in Figure 2 are expressed as:

(16) $$E_{i}(\xi )=\frac{E_{M}E_{A}}{\xi (E_{A}-E_{M})+E_{M}}\;(i=F,\;R)$$

where *E_F_* and *E_R_* are the loading and unloading elastic moduli, respectively, in the case of an incomplete phase transformation.

Similar to the major loop, the stresses of the turning points of the subloop shown in Figure 2 are the critical stress *σ_Ms_* upon loading and the critical stress *σ_As_* under unloading, respectively. This definition of turning point is the same as that in the literature [49,53].

Referring to the experimental results of the SMA material [54,55,56], two points need to be demonstrated here for the subloop characterized in this paper. One point is that the subloop is a closed hysteresis curve that returns to its initial point under unloading or loading. For instance, here the subloop in Figure 2 comes back to its initial point O′ under unloading. The other point is that the elastic modulus of subloop in the SMA wire gradually decreases along with the increase of the strain, as shown in the literature [56]. Sometimes the extension lines of the loading path O′-A′, the unloading path B′-D′, the path O-A in the austenitic phase and the path C-D in the martensitic phase can be roughly regarded to intersect at the same point. The above two points make the normal stress-strain relationship model presented in this paper different from the Motahari model and more consistent with the experimental results.

Based on the elastic moduli, *E_F_* and *E_R_*, obtained by Equation (16), the critical strain values in the subloop can be obtained as follows:

(17) $$\varepsilon_{M_{s}}^{\prime }=\varepsilon_{\min}^{\prime }+\frac{\sigma_{M_{s}}-\sigma_{\min}^{\prime }}{E_{F}}$$

(18) $$\varepsilon_{A_{s}}^{\prime }=\varepsilon_{\max}^{\prime }+\frac{\varepsilon_{A_{s}}-\varepsilon_{\max}^{\prime }}{E_{R}}$$

where $\varepsilon_{M_{s}}^{\prime }$ and $\varepsilon_{A_{s}}^{\prime }$ are the strains at point A′ and point D′, respectively. $\sigma_{\min}^{\prime }$ and $\sigma_{\max}^{\prime }$ are the minimum stress and the maximum stress in the subloop, respectively.

Therefore, four piecewise paths of the subloop are given as follows:

Path O′- A′:

(19) $$\sigma =\sigma_{\min}^{\prime }+E_{F}(\varepsilon -\varepsilon_{\min}^{\prime })$$

Path A′- B′:

(20) $$\sigma =\sigma_{M_{s}}+\frac{\sigma_{M_{f}}-\sigma_{M_{s}}}{\varepsilon_{M_{f}}-\varepsilon_{M_{s}}^{\prime }}(\varepsilon -\varepsilon_{M_{s}}^{\prime })$$

Path B′- D′:

(21) $$\sigma =\sigma_{\max}^{\prime }+E_{R}(\varepsilon -\varepsilon_{\max}^{\prime })$$

Path D′- O′:

(22) $$\sigma =\sigma_{A_{s}}+\frac{\sigma_{\min}^{\prime }-\sigma_{A_{s}}}{\varepsilon_{\min}^{\prime }-\varepsilon_{A_{s}}^{\prime }}(\varepsilon -\varepsilon_{A_{s}}^{\prime })$$

Let us consider the qualitative similarity between the experimental shear stress-strain relationship and the stress-strain relationship in the SMA material [57]. One can use the shear stress and strain to replace the normal stress and strain in Equations (1)–(22). Correspondingly, the material parameters in the shear stress-strain model can be determined directly from those in the normal stress-strain constitutive model, and the calculation formulas of the parameters are shown in Table 1 [35,58], in which *ν* is Poisson’s ratio and equals 0.33.

### 2.2. Constitutive Model of an SMA Helical Spring

**Figure 3:**
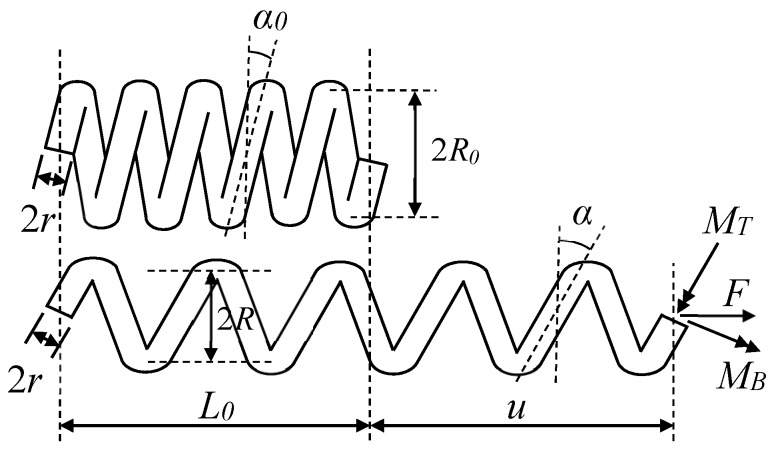
Schematic of a shape memory alloy (SMA) helical spring.

When an SMA helical spring undergoes a large axial deformation, the cross section of the spring wire has to resist a large bending moment in addition to the torque. To consider the influence of the bending moment on the spring, a two-dimensional mechanical model is adopted [59]. Note that if the ratio between coil and wire radii is small, a distortion of the otherwise symmetric strain distribution in the cross section of wire will occur. However, Mirzaeifar et al. [39] found that it did not have a significant impact on the force-displacement relationship of the SMA spring. For example, an SMA helical spring subjected to an axial force *F* is shown in Figure 3. It is assumed that for the SMA spring, the initial coil radius is *R*_0_, the wire radius is *r*, the initial length is *L*_0_, and the number of coils is *N*. Then, the initial pitch angle $\alpha_{0}$ can be obtained as

(23) $$\alpha_{0}=\arctan\left(L_{0}/\left(2\pi NR_{0}\right)\right)$$

In addition, the total length *L*, the coil radius *R*, and the pitch angle α of the deformed spring are calculated by the following formulas.

(24) $$L=\sqrt{L_{0}^{2}+{\left(2\pi NR_{0}\right)}^{2}}$$

(25) $$R=\frac{R_{0}\cos\alpha }{\cos\alpha_{0}}$$

(26) $$\alpha =\arcsin\left(\frac{u}{L}+\sin\alpha_{0}\right)$$

where *u* is the longitudinal displacement of the spring. 

**Figure 4:**
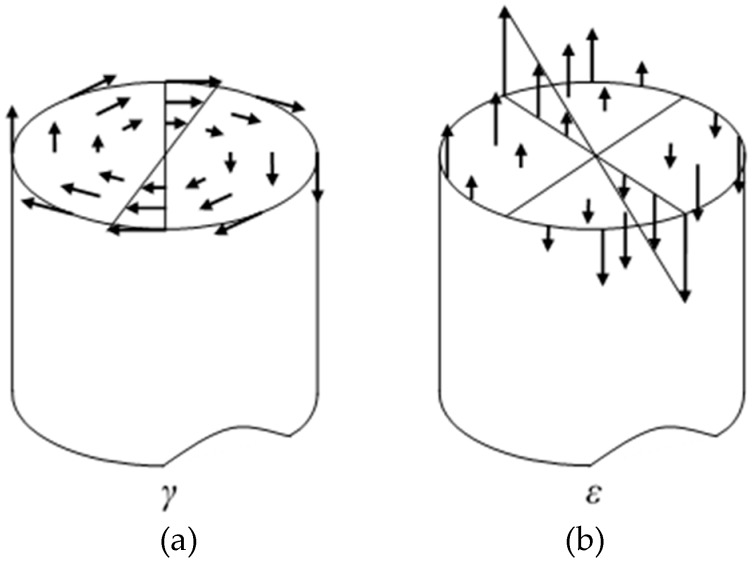
Schematic of the distributions of the shear strain *γ* and normal strain *ε* on the cross section of wire for (**a**) shear strain and (**b**) normal strain.

The bending moment and torque applied on the cross section of the wire will produce the normal and shear strain on the section, and the distributions of the normal and shear strain are plotted in Figure 4 [60]. Note that the coupling effect of tension-torsion on the distribution of shear and normal strains is not considered in this paper, as assumed in the literature [35,58]. However, the bending moment on the cross section of the wire actually will lead to the nonlinearity of the distribution of shear and normal strains, which can be verified through the experiments shown in the literature [61]. Comparing the experimental results in the literature [61] with those obtained in Section 3 of this paper, it is found that the degree of phase transformation of the SMA springs tested in this paper is weaker than that of the spring shown in the literature, which means the nonlinearity of the distribution of strains on the cross section of the wire is not strong for the springs manufactured in this paper. Therefore, for simplicity, the distributions of the normal and shear strains are assumed as linear here. However, in the case of the strong coupling of tension-torsion, the nonlinear distribution model of the strains have to be developed. 

**Figure 5:**
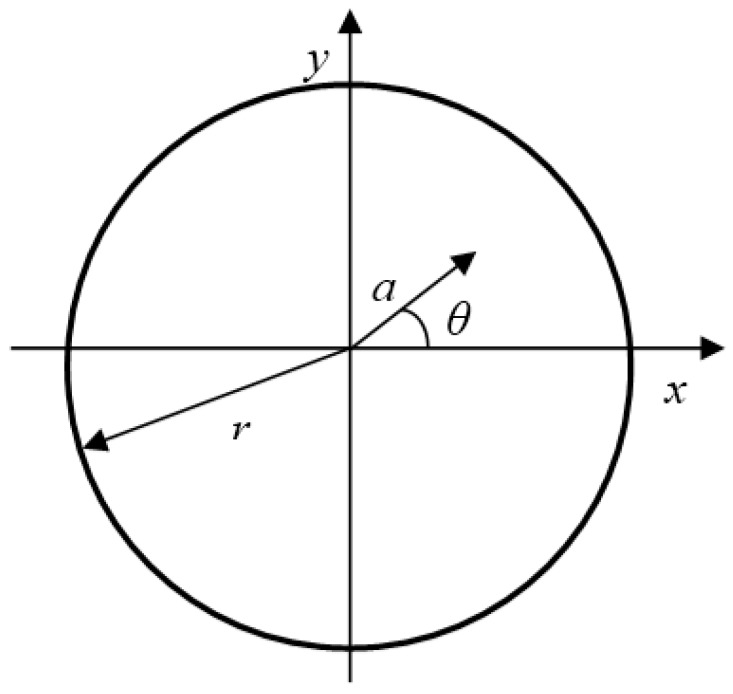
Schematic diagram of the cross section of the wire.

Figure 5 shows the Cartesian and radial coordinate systems of a cross section. The distribution of the normal strain in the Cartesian system is written as

(27) $$\begin{array}{l} \varepsilon \left(y\right)=y\left(\frac{\cos^{2}\alpha_{0}}{R_{0}}-\frac{\cos^{2}\alpha }{R}\right)\\ \;=\frac{y}{R_{0}}\cos\alpha_{0}\left(\cos\alpha_{0}-\cos\alpha \right) \end{array}$$

The distribution of the shear strain in the radial coordinate system is expressed as

(28) $$\begin{array}{l} \gamma \left(a\right)=a\left(\frac{\sin\alpha \cos\alpha }{R}-\frac{\sin\alpha_{0}\cos\alpha_{0}}{R_{0}}\right)\\ \;=\frac{a}{R_{0}}\cos\alpha_{0}\left(\sin\alpha -\sin\alpha_{0}\right) \end{array}$$

where *y* is a Cartesian coordinate perpendicular to the neutral axis of the spring, and *a* is the radial coordinate. 

Using Equations (27) and (28), and considering the stress-strain relationship Equations (5)–(8), the distribution of normal stress on the wire cross section can be obtained. Correspondingly the distribution of shear stress can also be determined by using the parameters in the shear stress-strain model shown in Table 1. Then the torque *M_T_* and the bending moment *M_B_* applied on the cross section can be calculated by

(29) $$M_{T}=\int_{0}^{r}\int_{-\pi }^{\pi }\tau \left(\gamma \left(\theta ,a\right)\right)a^{2}d\theta da$$

(30) $$M_{B}=\int_{0}^{r}\int_{-\pi }^{\pi }\sigma \left(\varepsilon \left(\theta ,a\right)\right)a^{2}\sin\theta d\theta da$$

where *θ* is the angular coordinate related to the radial coordinate *a*, as shown in Figure 5. 

Considering the cross section of the spring in Figure 3 and the effects of the torsional moment *M_T_* and the bending moment *M_B,_* a force equilibrium equation along the axial direction of the spring can be set up as

(31) $$\begin{array}{l} F=M_{T}\frac{\cos\alpha }{R}+M_{B}\frac{\sin\alpha }{R}\\ \;=\frac{\cos\alpha_{0}}{R_{0}}\left(M_{T}+M_{B}\tan\alpha \right) \end{array}$$

Following the above-mentioned steps, the force-displacement relationship of major loop for an SMA helical spring can be obtained. In a similar manner, using Equations (19)–(22) to replace Equations (5)–(8), the force-displacement relationship of subloops can also be determined. It should be emphasized that a series of experiments for a NiTi thin-walled tube implemented by Mehrabi et al. [62] shows an obvious effect of tension-torsion coupling. For example, a simple tension test will induce an out-of-plane shear strain in the thin-walled tube. Before that, if a torsion is applied to the tube, the induced shear strain by the tension will change the distribution of shear strain on cross section under the torsion. However, when one takes a close look at the simple tension test, it can be easily derived that by integrating all shear stress related to the distributed out-of-plane shear strain, the shear force on the cross section of the tube can be obtained, and it should equal to zero. More than that, using Equation (29), the torque on the cross section is also zero. According to this finding, it seems reasonable to consider the effects of bending and torsion on the SMA spring respectively. 

## 3. Experiments and Results

### 3.1. Tension Tests of SMA Helical Springs

**Figure 6:**
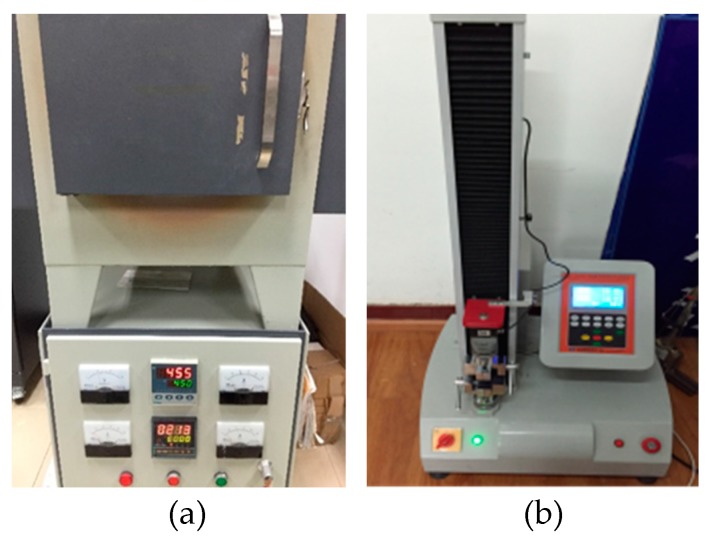
Heating and tension test equipment: (**a**) the oven; (**b**) the tensile test machine.

**Figure 7:**
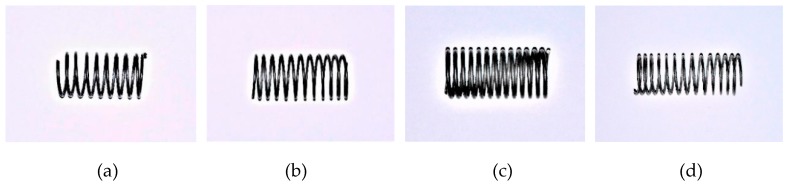
SMA helical spring specimens: (**a**) SMA-S1; (**b**) SMA-S2; (**a**) SMA-S3; (**b**) SMA-S4.

The Nitinol SMA wires used to train the springs were produced by Jiangyin Fasten Peier New Materials Technology Co. Ltd. in Jiangyin, China. The Ni content of the material is 50.8%. To fabricate the SMA helical springs, two types of SMA wires with diameters of 1 mm and 0.8 mm were used. During the fabrication of the SMA helical springs, thermal processing was employed. First, an SMA alloy wire was tightly wound on a screw rod. Then, the screw rod with the SMA wire was heated in an oven, as shown in Figure 6a. The heating temperature was 450 °C, and the heating lasted 60 min. After heating, the SMA wire was cooled in water. In this way, the four spring specimens made from the two types of wires, which are shown in Figure 7, are trained and are denoted by SMA-S1, SMA-S2, SMA-S3 and SMA-S4, respectively. The coil diameters of the SMA-S1, SMA-S2, SMA-S3 and SMA-S4 are 12.8 mm, 12.2 mm, 11 mm and 11.2 mm, respectively. The designed geometrical parameters of the SMA helical spring specimens and the material properties of the SMA wires obtained by material experiments are shown in Table 2 and Table 3, respectively.

**Figure 8:**
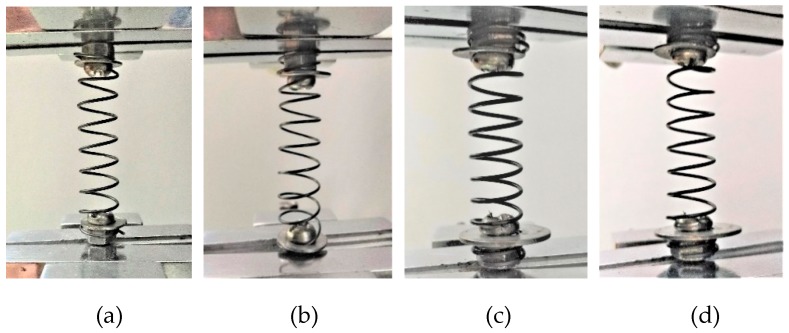
Tension tests of the SMA helical spring specimens: (**a**) SMA-S1; (**b**) SMA-S2; (**c**) SMA-S3; (**d**) SMA-S4.

A series of tension tests were carried out on the two SMA spring specimens using an HD-B609B-S-type material test machine, as shown in Figure 6b. For the machine, the maximum tensile force is 500 N, and the maximum stroke is 1000 mm. The tensile force has a precision of 0.001 N, and the precision of the controlled displacement reaches 0.01 mm. The maximum testing rate is 300 mm/min. In this paper, the loading of the spring specimens is controlled by the displacement, and the specific loading cases are shown in Table 4 [63]. In Table 4, the displacement amplitudes are given to clearly show the force-displacement curves of major loop and subloops in SMA springs. The applied testing rate is 150 mm/min, and the number of coils is 7 for both spring specimens. The environmental temperature of the tests was 25 °C. The loading states of the spring specimens are shown in Figure 8.

### 3.2. Testing Results

#### 3.2.1. Cyclic Loading with Variable Amplitudes

**Figure 9:**
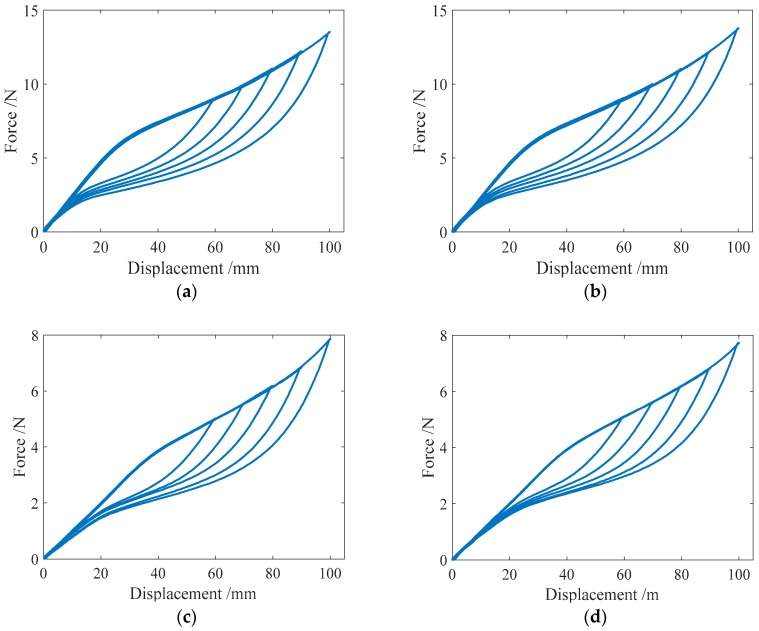

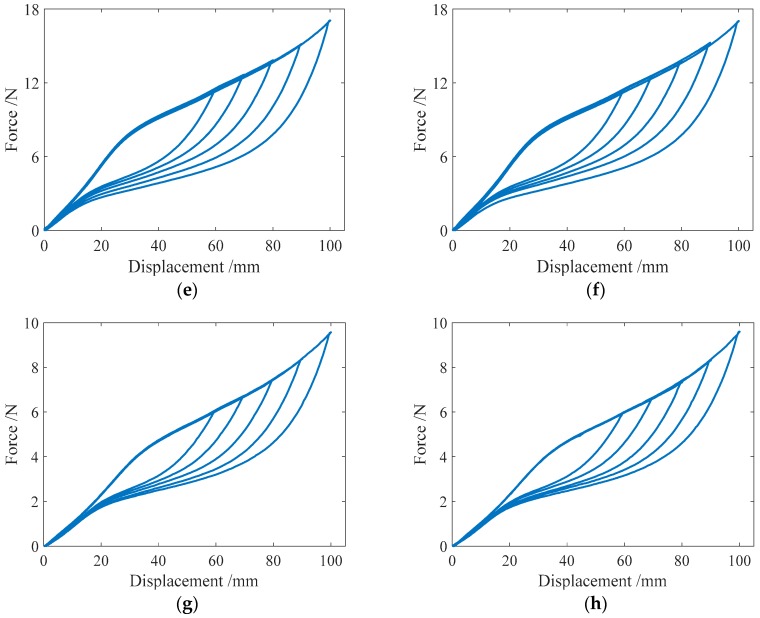
Force-displacement relationship curves for the spring specimens under cyclic loading with variable amplitudes: (**a**) SMA-S1 in Case I; (**b**) SMA-S1 in Case II; (**c**) SMA-S2 in Case I; (**d**) SMA-S2 in Case II; (**e**) SMA-S3 in Case I; (**f**) SMA-S3 in Case II; (**g**) SMA-S4 in Case I; (**h**) SMA-S4 in Case II.

Cyclic loading tests were carried out on the specimens SMA-S1, SMA-S2, SMA-S3 and SMA-S4 with different displacement amplitudes. The conducted loading cases were Case I and Case II, as listed in Table 4. The test results of the force-displacement relationships for the four spring specimens are plotted in Figure 9. Figure 9 shows that as the displacement amplitude of loading increases, the force-displacement hysteresis curves or major loops for the SMA helical springs broaden, which indicates that more energy dissipation occurs. Meanwhile, the residual displacements of the four spring specimens are close to zero after complete unloading. This phenomenon illustrates that the springs have a perfect recentering ability. It is worth mentioning that the force-displacement curve or major loop of each specimen in Case I is basically the same as that of the specimen in Case II when the displacement amplitudes of loading are identical. These results demonstrate that if the displacement amplitudes of loading are identical, the loading and unloading paths of the major loops of the SMA helical springs are stable for different cases of loading.

#### 3.2.2. Complex Single-Cycle Loading

**Figure 10:**
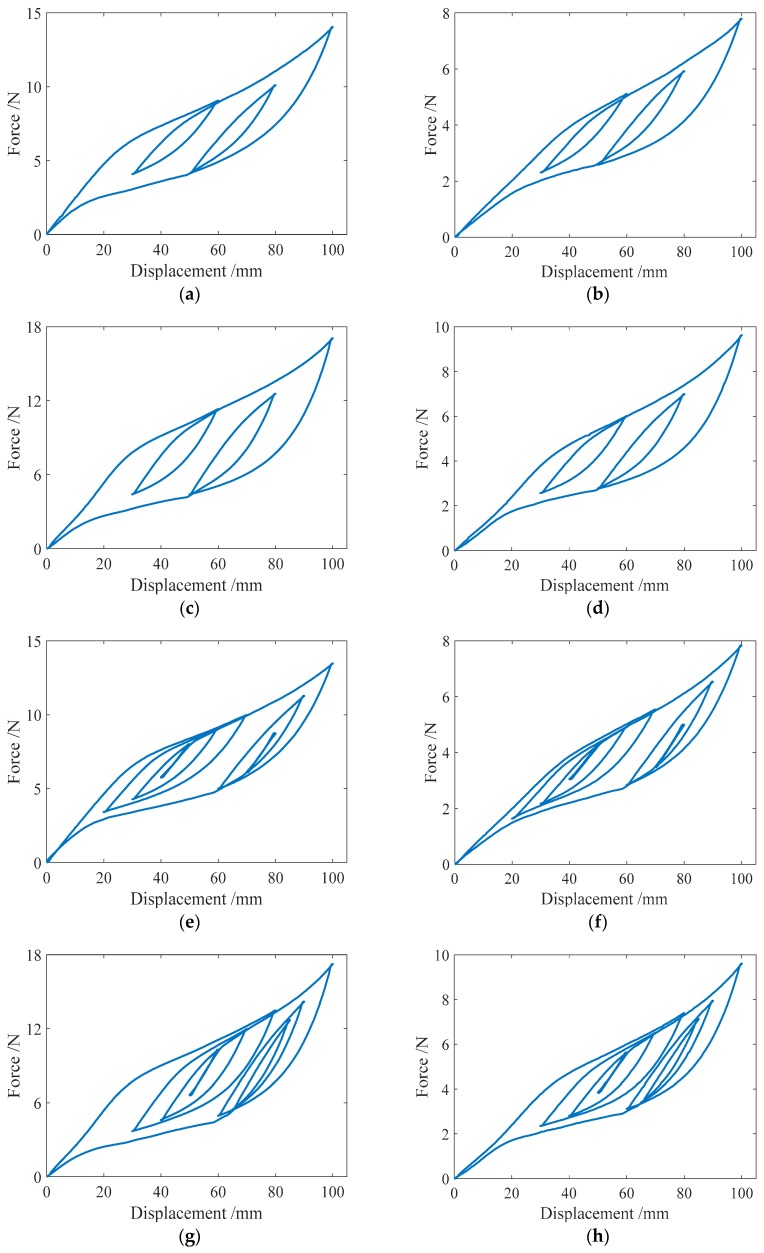
Force-displacement relationship curves of the spring specimens under complex single-cycle loading:(**a**) SMA-S1 in Case III; (**b**) SMA-S2 in Case III; (**c**) SMA-S3 in Case III; (**d**) SMA-S4 in Case III; (**e**) SMA-S1 in Case IV; (**f**) SMA-S2 in Case IV; (**g**) SMA-S3 in Case IV; (**h**) SMA-S4 in Case IV.

To produce the subloops in the force-displacement relationship curve of an SMA helical spring, the complex single-cycle loading tests, in which the loading Case III and IV shown in Table 4 are included, are specifically designed in this paper. The loading style of Case IV is more complex than that of Case III, and the two cases have the same maximum displacement amplitude. The test curves of the force-displacement relationship for the four specimens in the two loading cases are shown in Figure 10. From Figure 10a–d, it is found that for loading Case III, two single-cycle subloops appear in the force-displacement curves of the spring specimens SMA-S1, SMA-S2, SMA-S3 and SMA-S4. These subloops are basically closed loops where the starting point and the ending point are the same. In addition, the springs almost have no residual deformations after complete unloading. For Case IV, the force-displacement curves of the four spring specimens are plotted in Figure 10e–h, from which it can be observed that for the specimens SMA-S1, SMA-S2, SMA-S3 and SMA-S4, the multicycle subloops occur in the major loops of the force-displacement curves. The characteristics of these subloops are identical to those in Case III. The perfect recentering ability of the two specimens is also observed in Case IV. Furthermore, for Cases III and IV, the major loops of specimen SMA-S1 are almost the same, which means that the style of loading does not change the mechanical characterizations of the SMA springs trained in this paper. The same conclusion can be obtained from the results of the specimens SMA-S2, SMA-S3 and SMA-S4.

### 3.3. Comparison of the Numerical and Experimental Results

**Figure 11:**
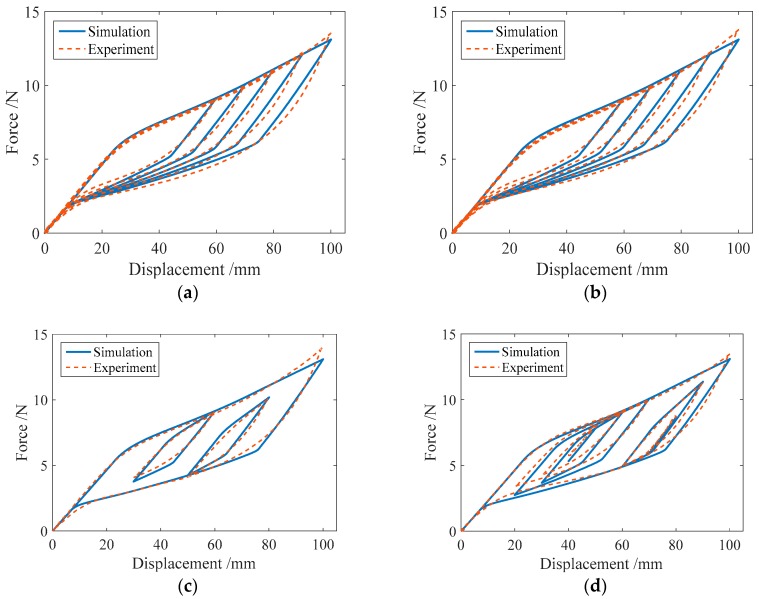
Simulation results of the force-displacement relationship of the specimen SMA-S1 in different loading cases: (**a**) Case I; (**b**) Case II; (**c**) Case III; and (**d**) Case IV.

In this section, the proposed mechanical model for the SMA springs will be used to simulate the hysteretic behavior of SMA helical springs under different load cases. The numerical results of the force-displacement relationship for specimen SMA-S1 in four loading cases are shown in Figure 11. For comparison, the corresponding experimental results for specimen SMA-S1 are also plotted in Figure 11.

From Figure 11, it can be observed that regardless of cyclic loading with variable amplitudes or complex cyclic loading, the simulation results of the major loops of specimen SMA-S1 agree well with the experimental results. Meanwhile, for the single-cycle and multicycle subloops, the simulation results are very consistent with the experimental results.

**Figure 12:**
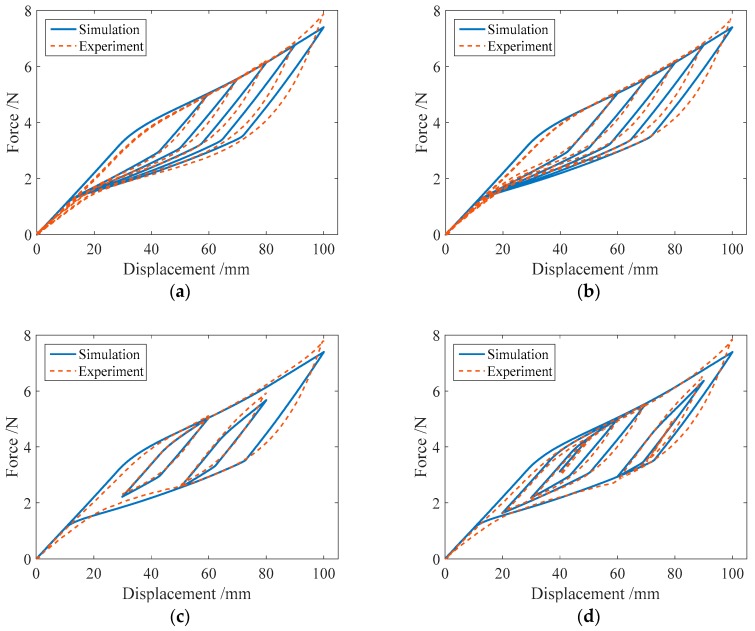
Simulation results of the force-displacement relationship of specimen SMA-S2 in different loading cases: (**a**) Case I; (**b**) Case II; (**c**) Case III; (**d**) Case IV.

In the same way, the numerical simulation results of the force-displacement relationship of specimen SMA-S2 are plotted in Figure 12. Figure 12 shows that in different loading cases, the simulation results of the force-displacement relationship of specimen SMA-S2 are in good agreement with the experimental results. These comparisons demonstrate the effectiveness of the proposed mechanical model for SMA springs.

**Figure 13:**
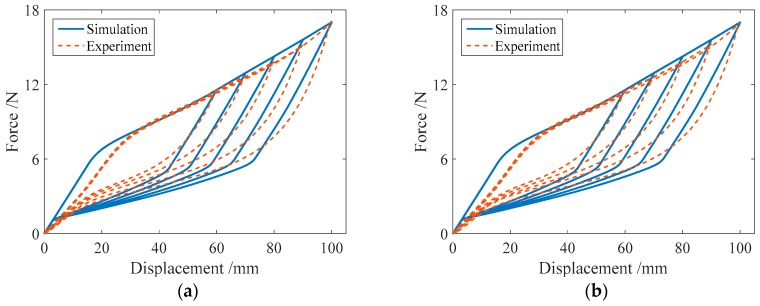

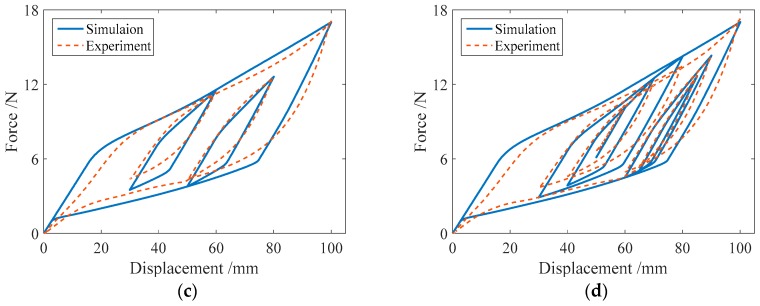
The simulation results of the force-displacement relationship of specimen SMA-S3 in different loading cases: (**a**) Case I; (**b**) Case II; (**c**) Case III; (**d**) Case IV.

Figure 13 shows the numerical results of the force-displacement relationship for specimen SMA-S3 in four loading cases. It is found from Table 2 that the specimen SMA-S3 has the same wire radius as the specimen SMA-S1, and the coil diameter of specimen SMA-S3 is less than that of specimen SMA-S1. In this situation, it is observed from Figure 13 that compared with specimen SMA-S1, the simulation force-displacement curves of specimen SMA-S3 are a little away from the corresponding experimental force-displacement curves. This finding indicates that the ratio of coil to wire radii has an influence on the force-displacement relationship in SMA springs. 

**Figure 14:**
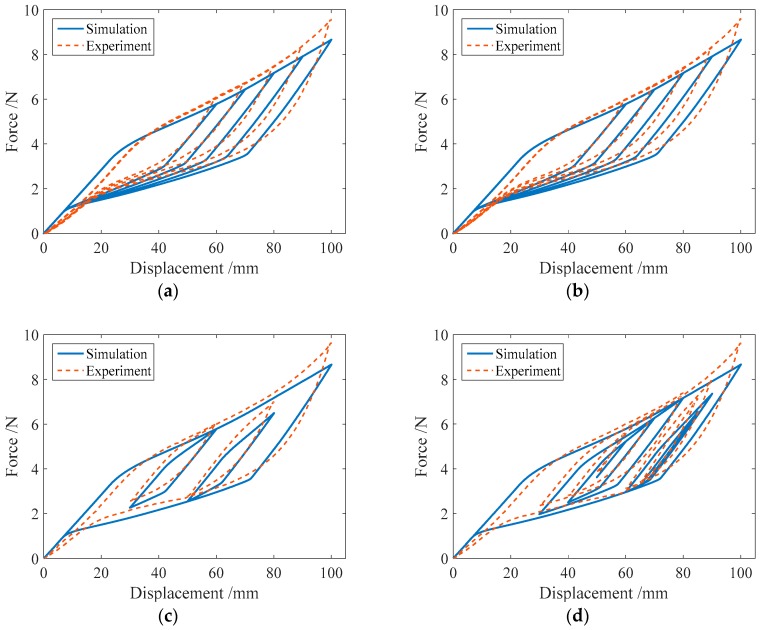
The simulation results of the force-displacement relationship of specimen SMA-S4 in different loading cases: (**a**) Case I; (**b**) Case II; (**c**) Case III; (**d**) Case IV.

The simulation results of the force-displacement relationship for specimen SMA-S4 in four loading cases are plotted in Figure 14. From Table 2, it is known that the ratio of coil to wire radii of specimen SMA-S4 is less than that of specimen SMA-S2. In this situation, it is found from Figure 14 that compared with specimen SMA-S2, the simulation force-displacement curves of specimen SMA-S4 are also a little away from the corresponding experimental force-displacement curves. This finding is similar to that from Figure 13.

**Figure 15:**
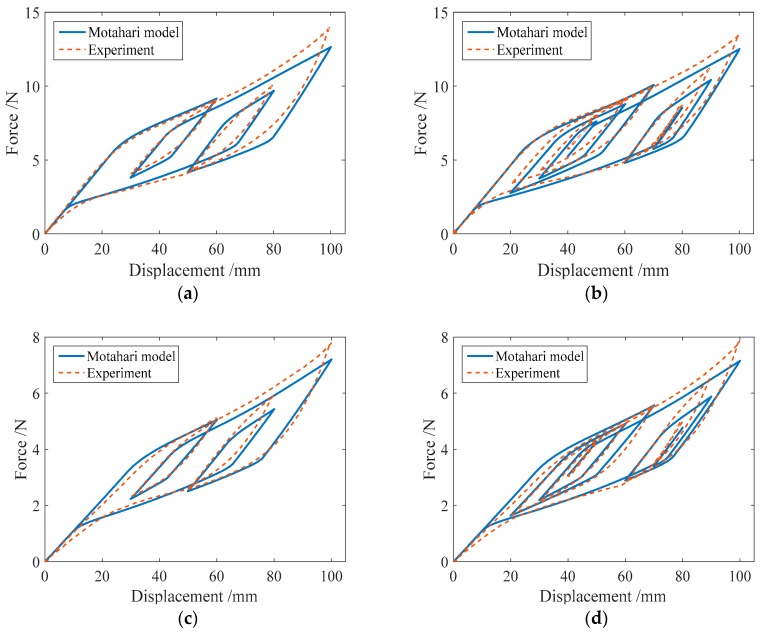

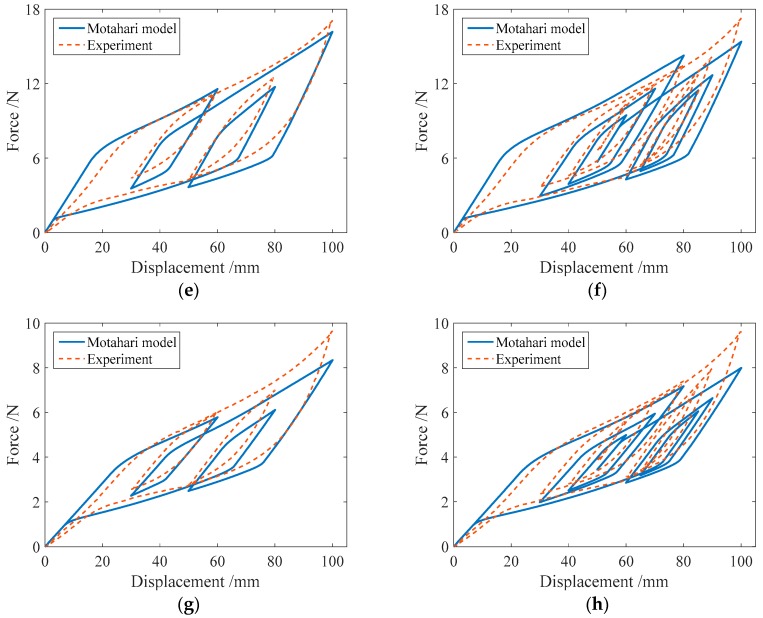
The numerical simulation results of the force-displacement relationship of the spring specimens from the Motahari model in the III and IV loading cases: (**a**) SMA-S1 in Case III; (**b**) SMA-S1 in Case IV; (**c**) SMA-S2 in Case III; (**d**) SMA-S2 in Case IV; (**e**) SMA-S3 in Case III; (**f**) SMA-S3 in Case IV; (**g**) SMA-S4 in Case III; (**h**) SMA-S4 in Case IV.

In addition, to illustrate the effect of the improvement with the proposed model, the numerical results of the force-displacement relationship of the SMA springs based on the 1D Motahari model of SMA material are plotted in Figure 15. In the simulation, four spring specimens SMA-S1, SMA-S2, SMA-S3 and SMA-S4 are used, and the III and IV loading cases listed in Table 4 are considered. From Figure 15, it can be found that when the loading displacement is not large, the numerical results from the Motahari model exhibit good agreement with the experimental results. As the controlled loading displacement increases, the simulated force-displacement curves, including the major loops and subloops, obviously deviate from the experimental results. Figure 15 also shows that the subloops from the Motahari model are not as accurate as those from the proposed model. Here, for the subloops, the starting points generally do not coincide with the end points, differing from the experimental results. As a whole, the numerical results of the proposed model, which are shown in Figure 11, Figure 12, Figure 13 and Figure 14, are closer to the corresponding experimental results than those of the Motahari model, demonstrating the improvement of the proposed model.

**Figure 16:**
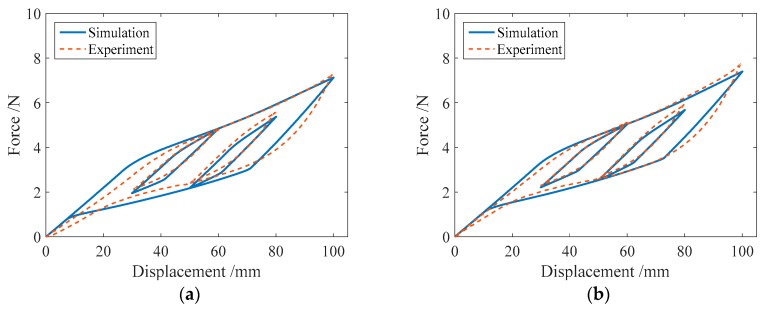

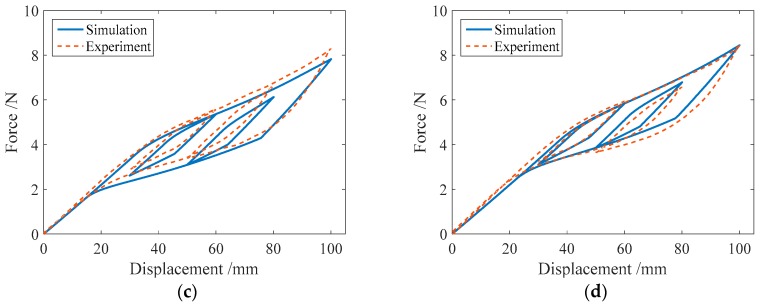
The simulation results of the force-displacement relationship of specimen SMA-S2 at different temperatures: (**a**) 22 °C; (**b**) 25 °C; (**c**) 30 °C; (**d**) 40 °C.

To illustrate the effect of temperature on the force-displacement relationship in the SMA springs, the force-displacement relationship of specimen SMA-S2 at different temperatures is studied, and the simulation results are plotted in Figure 16. From Figure 16, it is found that when the temperature varies from 22 °C to 40 °C, the simulation results from the proposed model are in a good agreement with those from experiments. The major loop becomes small along with the increase of temperature. Similar phenomena can also be found in the literature [44]. 

## 4. Conclusions

This paper proposes a new simplified mechanical model to simulate the force-displacement relationship of helical superelastic SMA springs. The new model inherits the advantage of simple computation of the multilinear constitutive model and improves the 1D Motahari constitutive model of the superelastic SMA material to better fit the experimental results. Furthermore, the established force-displacement relationship considers the influences of not only the torque but also the moment on the cross sections of the SMA wires. The experimental studies include a series of tension tests on four NiTi helical spring specimens in four loading cases. The experimental results show that the SMA spring specimens exhibit stable superelasticity and a very good recentering ability. In complex loading cases, the force-displacement curves for the SMA springs mainly consist of major loop and subloops. Compared with the force-displacement curves simulated by the Motahari model, those simulated by the proposed model can better approximate the experimental results. When the temperature changes in some range, the proposed model still works very well. In summary, the proposed model predicts the force-displacement relationship well for helical SMA springs. Moreover, this new model is very helpful for using SMA springs to design precise displacement sensors or force sensors.

## Figures and Tables

**Table 1:** The formulas for the material parameters in the shear stress-strain model.

Calculation Formula	Parameter
*G_A_* = *E_A_*/2(1 + *ν*)	Shear modulus of austenite
*G_M_* = *E_M_*/2(1 + *ν*)	Shear modulus of martensite
*τ_Ms_* = *σ_Ms_*/$\sqrt{3}$	Starting shear stress of the martensitic transformation
*τ_Mf_* = *σ_Mf_*/$\sqrt{3}$	Final shear stress of the martensitic transformation
*τ_As_* = *σ_As_*/$\sqrt{3}$	Starting shear stress of the austenitic transformation
*τ_Af_* = *σ_Af_*/$\sqrt{3}$	Final shear stress of the austenitic transformation
*γ_L_* = *ε_L_*	Maximum residual shear strain

**Table 2:** Geometrical parameters of the SMA helical spring specimens.

Specimen	*R*_0_ (mm)	*r* (mm)	*L*_0_ (mm)	*N*
SMA-S1	6.4	0.5	22	7
SMA-S2	6.1	0.4	19	7
SMA-S3	5.5	0.5	12	7
SMA-S4	5.6	0.4	13	7

**Table 3:** Material properties of the SMA wires.

Specimen	*E*_A_ (GPa)	*E*_M_ (GPa)	*C*_A_ (MPa/K)	*C*_M_ (MPa/K)	*M*_S_ (K)	*A*s (K)	*A*_f_ (K)	*σ_s_* (MPa)	*σ_f_* (MPa)	*ε_L_*
SMA-S1	72	60	11.0	6.8	269	280	290	99	527	0.037
SMA-S2	72	64	11.0	7.6	268	278	288	96	582	0.032
SMA-S3	72	60	11.0	6.8	269	280	290	99	527	0.037
SMA-S4	72	64	11.0	7.6	268	278	288	96	582	0.032

**Table 4:** Loading cases for the SMA spring specimens.

Case	Displacement Amplitudes (mm)	Loading History
I	60, 70, 80, 90 and 100	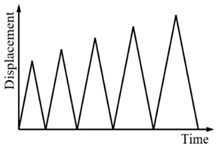
II	100, 90, 80, 70 and 60	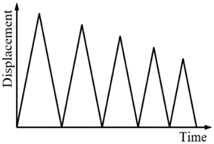
III	60, 30, 100, 50 and 80	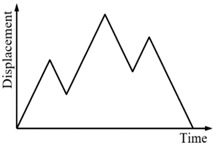
IV	80, 30, 70, 40, 60, 50, 100, 60, 90, 65 and 85